# Severe acute kidney injury with anuria induced by hypokalemia requiring hemodialysis: a case study

**DOI:** 10.1186/s12882-025-03973-z

**Published:** 2025-03-24

**Authors:** Seong-Wook Lee, Man-Hoon Han, Mee-Seon Kim, Yong-Jin Kim, You Hyun Jeon, Hee-Yeon Jung, Ji-Young Choi, Jang-Hee Cho, Sun-Hee Park, Chan-Duck Kim, Yong-Lim Kim, Jeong-Hoon Lim

**Affiliations:** 1 Present Address: Division of Nephrology, Department of Internal Medicine, School of Medicine, Kyungpook National University, Kyungpook National University Hospital, Daegu, South Korea; 2 Department of Pathology, School of Medicine, Kyungpook National University, Kyungpook National University Hospital, Daegu, South Korea; 3 Department of Pathology, School of Dentistry, Kyungpook National University, Kyungpook National University Hospital, Daegu, South Korea

**Keywords:** Acute kidney injury, Anuria, Hypokalemic nephropathy, Kidney biopsy, Vacuolization, WNK bodies

## Abstract

**Background:**

Hypokalemia can result from various causes, with diarrhea being one of the most common. Although rare, chronic hypokalemia can lead to severe acute kidney injury (AKI) that requires dialysis. Therefore, this case study aims to investigate a patient with rectal cancer who, after concurrent chemoradiotherapy and ileostomy, developed chronic hypokalemia owing to prolonged diarrhea, leading to severe AKI with anuria.

**Case presentation:**

A 64-year-old man with a history of rectal cancer, ileostomy, and hypertension was admitted for severe AKI with anuria. He had developed severe hypokalemia due to chronic diarrhea. Despite having no prior kidney disease, his serum creatinine increased to 4.8 mg/dL, and potassium dropped to 2.2 mmol/L. Initial treatment included hemodialysis for anuric AKI with metabolic acidosis. A kidney biopsy revealed renal tubular vacuolization and With-no-lysine kinase (WNK) bodies in the distal tubules, which are characteristic of hypokalemic nephropathy. Potassium replacement therapy led to a gradual recovery of potassium levels and kidney function.

**Conclusion:**

This case highlights the importance of timely diagnosis and management of hypokalemic nephropathy through kidney biopsy.

## Background

Hypokalemia is a common electrolyte imbalance in clinical practice, resulting from potassium shifts into cells, excessive potassium loss, or, less frequently, inadequate intake [1]. In a prospective study of 322,046 hospitalized patients, 13.7% were found to have hypokalemia [2]. Clinical presentations vary depending on the cause, with hypokalemia manifesting as an acute, subacute, or chronic condition. Gastrointestinal and renal losses are the most frequent causes of hypokalemia [3].

Clinical symptoms of hypokalemia vary with severity and may include muscle weakness, cramps, paralysis, palpitations, and constipation [1]. Severe hypokalemia can lead to life-threatening complications, including fatal cardiac arrhythmias and respiratory failure [1, 4]. Hypokalemia is also a significant risk factor for worsening chronic kidney disease (CKD) and is associated with higher risks of mortality, hospitalization, and cardiovascular events in patients with CKD [5–8].

Chronic hypokalemia leads to intracellular potassium depletion, directly affecting renal tubular cells. Potassium loss in these cells can disrupt cellular function, particularly in the proximal and distal tubules, where potassium is essential for maintaining cell volume and ionic balance. This dysfunction can cause tubular injury, reduced reabsorption, and, eventually, tubular atrophy. Over time, these changes may lead to interstitial scarring, renal insufficiency, and medullary renal cyst formation [9]. Chronic hypokalemia is associated with a specific histopathological condition called chronic hypokalemic nephropathy, characterized by tubular vacuolar lesions in the epithelial cells [10–13]. Recent studies have also identified With-no-lysine kinase (WNK) bodies as punctate structures in the distal convoluted tubule (DCT). These bodies consist of WNK and Ste20-related proline-alanine-rich kinase (SPAK) proteins, which regulate the Na-Cl cotransporter (NCC) and are located in non-membrane-bound cytoplasmic regions [14, 15].

Chronic hypokalemia is often caused by malnutrition, laxative abuse, and diuretic use. While chronic diarrhea can lead to hypokalemic nephropathy, severe acute kidney injury requiring dialysis and accompanied by anuria is rare. Therefore, this study aims to present a case of hypokalemic nephropathy that led to severe acute kidney injury (AKI) with anuria requiring hemodialysis.

## Case presentation

In July 2024, a 64-year-old patient was referred to the emergency room (ER) with AKI and hypokalemia. The patient had experienced chronic diarrhea for several months and developed leg muscle weakness 1 week prior. In the days leading up to the ER visit, the condition of the patient worsened, resulting in an inability to walk.

In medical history of the patient includes a diagnosis of rectal cancer confirmed by endoscopic biopsy in March 2023 (16 months ago). He underwent concurrent chemoradiotherapy (CCRT) and had an ileostomy placed after rectal cancer surgery on July 2023. The patient’s preoperative serum creatinine and potassium levels of  0.8 mg/dL and 4.4 mmol/L, respectively, were within the normal range. During the 7 months following CCRT, the potassium level was in the normal range of 3.7 to 4.1 mmol/L, and from March 2024 (4 months ago), the potassium level was low at 2.5 to 3.1 mmol/L. The patient was also diagnosed with hypertension 3 years ago and prescribed valsartan 80 mg daily. No significant family history of kidney disease was observed, and the patient denies using diuretics, nonsteroidal anti-inflammatory drugs, or herbal medicines.

At admission, the patient had a body mass index of 23.7 kg/m², blood pressure of 140/99 mmHg, and body temperature of 36.4 °C. The initial 24 h urine volume was 205 mL. Physical examination revealed no abnormalities with no peripheral edema. Abdominal computed tomography (CT) scan showed normal size and shape of both kidneys, with no signs of obstructive nephropathy.

The laboratory analysis showed blood urea nitrogen and creatinine levels of 137 and 4.8 mg/dL, respectively. The serum electrolytes were as follows: sodium 140, potassium 2.2, and chloride 109 mmol/L, magnesium 2.5, calcium 8.3, phosphate 5.3, and uric acid 13.7 mg/dL. Hemoglobin was 13.5 g/dL, and platelet count was 238.0 × 10^9^/L, both within the normal range. However, the white blood cell count was elevated at 18.7 × 10^9^/L, and C-reactive protein was elevated to 7.8 mg/dL despite no evidence of infection on cultures and chest and abdominal CT scans. Serum protein and albumin levels were 8.3 and 4.1 g/dL, respectively. The biomarker for AKI, neutrophil gelatinase-A lipocalin (NGAL), was elevated at 2396.6 ng/mL. The venous blood gas analysis showed a pH of 7.22, anion gap of 18.8 mmol/L, pCO_2_ of 16.9 mmHg, and bicarbonate level of 7.4 mmol/L.

The urine potassium concentration was 21 mmol/L, with urine and serum osmolality measured at 339 and 302 mOsm/kg, respectively. The trans-tubular potassium gradient was 9.0. Serum and urine immunologic tests associated with glomerulonephritis, and viral markers, such as hepatitis and HIV were all negative. Urinalysis indicated microscopic hematuria (1+) and proteinuria (1+), with a spot urine protein-to-creatinine ratio of 1.12 g/g creatinine.

**Fig. 1:**
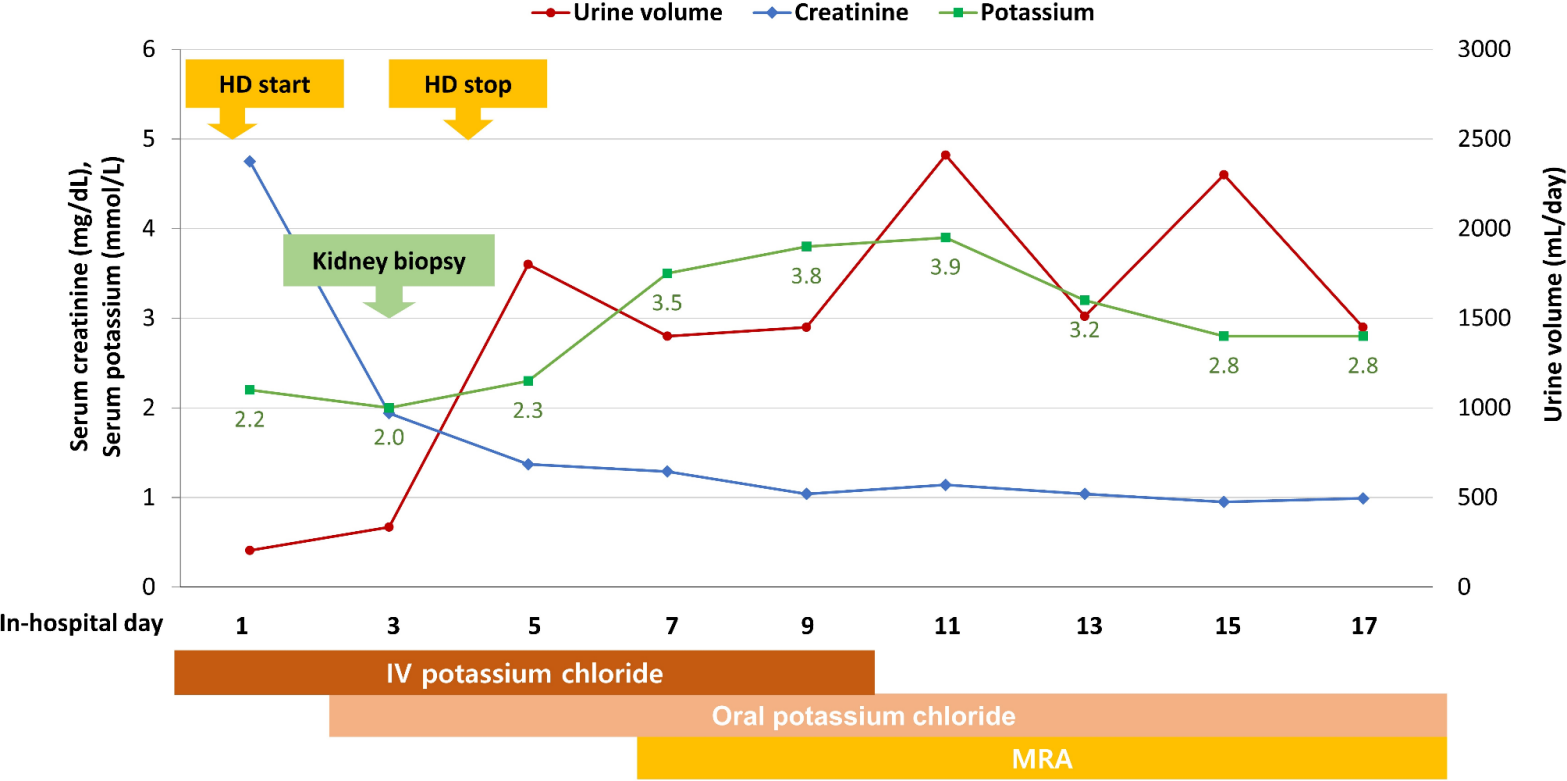
Changes in the clinical course of the patient. Abbreviations: HD, hemodialysis; MRA, mineralocorticoid receptor antagonist

**Fig. 2:**
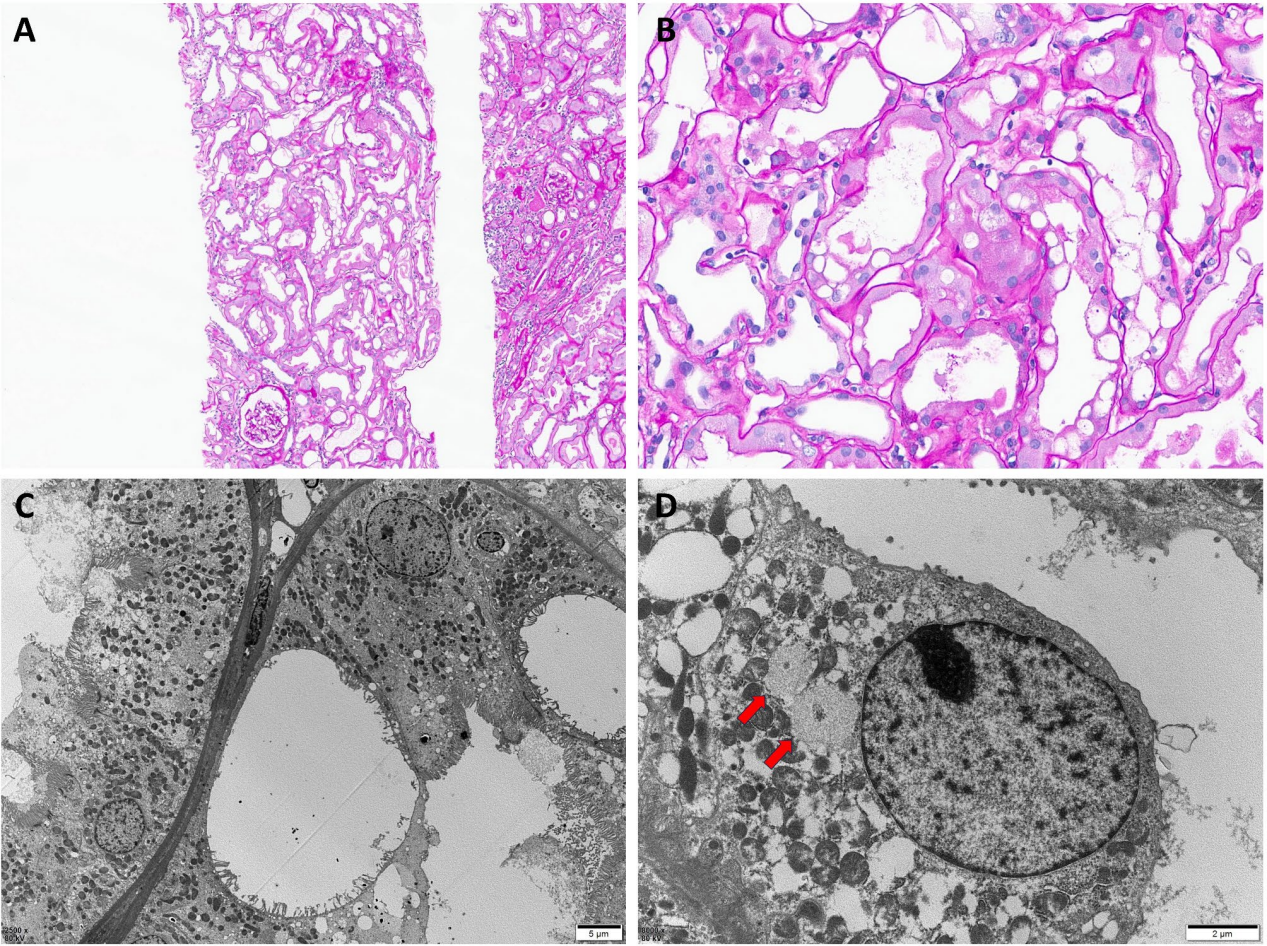
Histopathologic findings of kidney biopsy. (**A**) Diffuse vacuolization of the proximal tubules with acute tubular injury (Periodic Acid–Schiff; original magnification ×100). (**B**) Large vacuoles in proximal tubular epithelial cells with flattened tubular epithelium and dilated tubular lumen (Periodic Acid–Schiff; original magnification ×400). (**C**) Electron microscopy reveals intracytoplasmic, electron-lucent vacuoles in the proximal tubular epithelial cells (original magnification ×2500). (**D**) With no lysine kinase (WNK) bodies in the distal tubular epithelial cell (red arrows; original magnification ×8000)

On the first day of admission, hemodialysis was performed to manage oliguric AKI with metabolic acidosis (Fig. 1). A kidney biopsy was performed on day 3 to determine the underlying cause of AKI (Fig. 2). Light microscopy of the kidney biopsy revealed that none of the 11 glomeruli showed glomerulosclerosis, while mild tubular atrophy (6–25%), interstitial fibrosis (10–15%), and interstitial infiltration (10–25%) of lymphocytes and plasma cells were present (Fig. 2A). The periodic acid–Schiff stain revealed large vacuoles in the proximal tubular epithelial cells with flattened tubular epithelium and dilated tubular lumen (Fig. 2B). Immunofluorescence microscopy showed no significant abnormalities. Electron microscopy revealed normal epithelial cells and an intact glomerular basement membrane without unusual electron-dense deposits. However, large vacuoles were present in the proximal tubular epithelial cells (Fig. 2C), and WNK bodies were observed in the distal tubular epithelial cells (Fig. 2D).

The kidney biopsy confirmed hypokalemic nephropathy as the cause of the AKI. Treatment for hypokalemia included intravenous and oral potassium supplements, along with anti-diarrheal agents. To minimize potassium loss, the potassium-sparing diuretic medication (spironolactone) was also administered. During the course of treatment, the potassium levels of the patient gradually improved, leading to corresponding increases in kidney function and urine output. On the 17th day of admission, the patient was discharged with serum potassium and creatinine levels of 2.8 and 1.0 mg/dL, respectively, and a reduced NGAL level of 495.7 ng/mL. Furthermore, at a 3-month outpatient follow-up, kidney function remained stable, with potassium and creatinine levels of 4.5 mmol/L and 1.0 mg/dL, respectively.

## Discussion

This case report highlights the diagnostic and treatment considerations for severe hypokalemic nephropathy with anuria. The patient had normal baseline kidney function before undergoing ileostomy but developed chronic diarrhea post-surgery. Although the patient was seen regularly in oncology and surgical outpatient clinics, hypokalemia was not addressed, leading to severe AKI with anuria. This case underscores the importance of closely monitoring potassium levels and kidney function in patients with chronic diarrhea.

Conn J.W. and Johnson R.D. first used the term ‘‘kaliopenic nephropathy’’ to describe renal lesions, particularly tubular vacuolization, in patients with chronic hypokalemia [10, 16]. Histopathological findings from the kidney biopsy in this case aligned with these characteristics, further supporting the diagnosis. Tubular vacuolization typically impairs urine concentration but can be reversible with potassium repletion [17]. In this case, the patient presented with anuria—an atypical presentation of hypokalemia—but showed rapid improvement in kidney function following potassium replacement.

The exact mechanism by which hypokalemia causes kidney injury remains unclear; however, several mechanisms have been proposed. A previous study analyzing kidney biopsies from 40 patients with chronic hypokalemia found that 75% showed predominantly focal lymphocytic infiltration. Vacuoles may form in tubular cells, arterial smooth muscle, pericytes, and podocytes [18]. Other studies report that patients with chronic hypokalemic nephropathy can develop irreversible interstitial fibrosis due to diffuse chronic interstitial nephritis, which contributes to CKD progression [11, 12]. In hypokalemic rats, Reungjui et al. observed peritubular capillary loss, macrophage infiltration, and oxidative stress markers associated with fibrosis and impaired angiogenesis [7]. A separate rat study showed that chronic hypokalemia led to progressive tubulointerstitial injury, with elevated ammonia levels contributing to hypokalemic nephropathy by activating the alternative complement pathway [8]. Chronic hypokalemia is also associated with reduced renal blood flow and increased vascular resistance, driven by vasoactive mediators, such as angiotensin II, endothelin − 1, and thromboxane B2 [19, 20]. Additionally, Tsao et al. found that in hypokalemic rats, insulin-like growth factor-1 (IGF-1) trapping and overexpression of transforming growth factor-β (TGF-β) promoted cellular hypertrophy and fibrosis, which could lead to irreversible kidney damage if untreated [21]. However, in this case, the duration of hypokalemia was relatively short, approximately four months, resulting in mild interstitial fibrosis. The predominant finding was tubular vacuolization, which is known to be a reversible change [17], that allowed rapid recovery of kidney function following potassium supplementation.

The kidneys are crucial for maintaining electrolyte balance, with WNK kinases—a family of serine-threonine kinases—playing a key role in regulating salt, potassium, and pH levels [15]. Mutations in WNKs have been associated with familial hyperkalemic hypertension, which is characterized by hypertension and hyperkalemia [22]. The thiazide-sensitive NCC, found exclusively in the DCT, is activated by WNKs through a kinase cascade involving SPAK and OSR1 [23]. This cascade enhances sodium reabsorption in the DCT and reduces potassium excretion downstream [14, 24]. Thus, the WNK-SPAK/OSR1 pathway is essential for potassium homeostasis. In response to potassium deficiency, WNK complexes form large aggregates known as “WNK bodies.” These structures were first identified in hypokalemic rat models and later observed in human kidney biopsies from patients with chronic hypokalemia [14, 15]. The WNK-SPAK clusters, located in the cytoplasm near the nucleus, do not contain the typical components found in protein aggregates [14]. In this case, WNK bodies were observed in the distal tubules; however, their role in kidney injury is unclear and warrants further investigation.

Chronic diarrhea is a major cause of AKI, primarily through volume depletion [25]. In such cases, physicians typically focus on fluid replacement while waiting for recovery, often overlooking the potential role of electrolyte imbalances caused by diarrhea. This case is significant as it reports a rare case in which chronic hypokalemia lasting over four months led to tubular abnormalities and severe AKI with anuria. Although acute tubular injury, characterized by epithelial flattening and tubular dilation, can result from both volume depletion from chronic diarrhea and hypokalemic nephropathy [26], this case underscores the critical role of hypokalemia in AKI. It highlights the characteristic biopsy findings of hypokalemic nephropathy, such as tubular vacuolation and WNK bodies, and demonstrates a reversible clinical course with potassium supplementation.

## Conclusions

This case study demonstrates that chronic hypokalemia can lead to severe hypokalemic nephropathy with rapid kidney function deterioration. The extent of renal injury varies; however, in severe cases, such as this one, hemodialysis may be necessary. Histological findings, including tubular epithelial vacuolization and WNK bodies, aid in the diagnosis. Raising awareness of this condition is critical, as early diagnosis and intervention can prevent the progression of hypokalemic nephropathy to irreversible interstitial fibrosis and improve outcomes. Further research is needed to better understand the course and pathophysiology of the disease.

## Data Availability

The data generated and analyzed in this case are included in the manuscript.

## Declarations

***Clinical trial number***.

## Abbreviations

AKI: Acute kidney injury
CCRT: Concurrent chemoradiotherapy
CKD: Chronic kidney disease
DCT: Distal convoluted tubule
ER: Emergency room
IGF-1: Insulin-like growth factor-1
NCC: Na-Cl cotransporter
NGAL: Neutrophil Gelatinase-A lipocalin
SPAK: Ste20-related proline-alanine-rich kinase
TGF-β: Transforming growth factor-β
WNK: With-no-lysine kinase
